# (2*S*)-Methyl 2-(4-chloro­benzene­sulfon­amido)-4-(methyl­sulfan­yl)butanoate

**DOI:** 10.1107/S1600536809018297

**Published:** 2009-05-20

**Authors:** Tayyaba Syed, Shahid Hameed, Peter G. Jones

**Affiliations:** a Department of Chemistry, Quaid-i-Azam University, Islamabad 45320, Pakistan; b Institut for Anorganische und Analytische Chemie, Technische Universität Braunschweig, Hagenring 30, 38106 Braunschweig, Germany

## Abstract

The enanti­omerically pure title compound, C_12_H_16_ClNO_4_S_2_, contains a pyramidal N atom with an S—N bond length of 1.6306 (15) Å. Mol­ecules are linked to form chains parallel to the *a* axis by classical N—H⋯O hydrogen bonding involving a sulfonyl O atom, supported by three weak C—H⋯*X* inter­actions. (*X* = S, O).

## Related literature

For the applications of esters in industry and as inter­mediates in the synthesis of heterocycles, see: Akhtar *et al.* (2007 ▶, 2008 ▶); Kashif *et al.* (2008 ▶); Serwar *et al.* (2009 ▶); Syed *et al.* (2009 ▶).
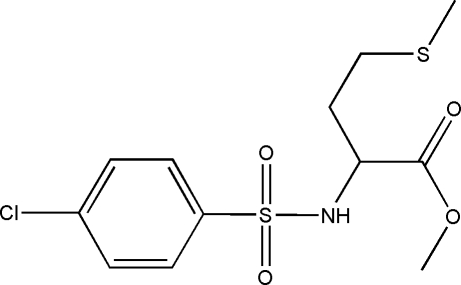


## Experimental

### 

#### Crystal data

C_12_H_16_ClNO_4_S_2_
*M* *_r_* = 337.83
Orthorhombic, 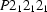

*a* = 5.1814 (3) Å
*b* = 12.6089 (8) Å
*c* = 23.2137 (13) Å
*V* = 1516.59 (16) Å^3^
*Z* = 4
Cu *K*α radiation
μ = 4.92 mm^−1^
*T* = 100 K
0.20 × 0.12 × 0.06 mm

#### Data collection

Oxford Diffraction Xcalibur Nova A diffractometer
Absorption correction: multi-scan (*CrysAlis Pro*; Oxford Diffraction, 2008 ▶) *T*_min_ = 0.548, *T*_max_ = 1.000 (expected range = 0.408–0.744)
14469 measured reflections
3093 independent reflections
3027 reflections with *I* > 2σ(*I*)
*R*_int_ = 0.033

#### Refinement

*R*[*F* ^2^ > 2σ(*F* ^2^)] = 0.025
*wR*(*F* ^2^) = 0.062
*S* = 1.04
3093 reflections
187 parameters
H atoms treated by a mixture of independent and constrained refinement
Δρ_max_ = 0.20 e Å^−3^
Δρ_min_ = −0.35 e Å^−3^
Absolute structure: Flack (1983 ▶), 1250 Friedel pairs
Flack parameter: 0.005 (12)

### 

Data collection: *CrysAlis Pro* (Oxford Diffraction, 2008 ▶); cell refinement: *CrysAlis Pro*; data reduction: *CrysAlis Pro*; program(s) used to solve structure: *SHELXS97* (Sheldrick, 2008 ▶); program(s) used to refine structure: *SHELXL97* (Sheldrick, 2008 ▶); molecular graphics: *XP* (Siemens, 1994 ▶); software used to prepare material for publication: *SHELXL97*.

## Supplementary Material

Crystal structure: contains datablocks I, global. DOI: 10.1107/S1600536809018297/bt2959sup1.cif

Structure factors: contains datablocks I. DOI: 10.1107/S1600536809018297/bt2959Isup2.hkl

Additional supplementary materials:  crystallographic information; 3D view; checkCIF report

## Figures and Tables

**Table 1:** Hydrogen-bond geometry (Å, °)

*D*—H⋯*A*	*D*—H	H⋯*A*	*D*⋯*A*	*D*—H⋯*A*
N—H01⋯O3^i^	0.81 (3)	2.30 (3)	3.1048 (18)	169 (3)
C2—H2⋯O1^ii^	1.00	2.66	3.637 (2)	166
C12—H12⋯O1^ii^	0.95	2.37	3.315 (2)	173
C3—H3*B*⋯O3^iii^	0.99	2.66	3.635 (2)	170
C5—H5*B*⋯S1^iv^	0.98	2.97	3.892 (2)	157
C5—H5*C*⋯S1^i^	0.98	2.88	3.665 (2)	138

Symmetry codes: (i) 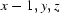; (ii) 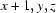; (iii) 
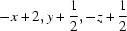
; (iv) 
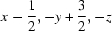
.

**Fig. 1.**
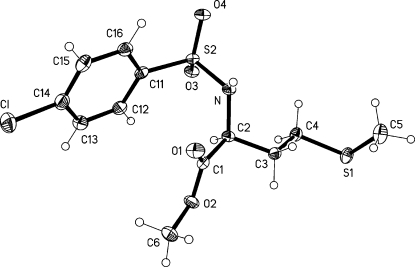
The molecule of the title compound. Ellipsoids correspond to 50% probability levels.

**Fig. 2.**
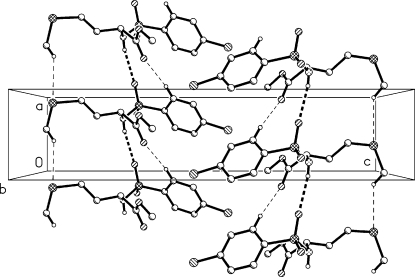
Packing diagram of the title compound, showing classical hydrogen bonds (thick dashed lines) and "weak" hydrogen bonds (thin dashed lines). H atoms not involved in these H bonds have been omitted. The interactions H2···O1 are omitted for clarity.

## supplementary crystallographic information

### Comment

Esters have attracted widespread attention due to their applications in industry and as intermediates in the synthesis of heterocycles (Syed *et al.*, 2009; Akhtar *et al.*, 2008, 2007; Serwar *et al.*, 2009). several types of pharmacological activities have also been associated with sulfonamides (Akhtar *et al.*, 2008, Kashif *et al.*, 2008). The title compound (I), a methionine derivative, was synthesized in our laboratory as an intermediate for onward conversion to 1,3,4-oxadiazole derivatives, and here we report its structure.

Molecular dimensions of (I) may be considered normal. The nitrogen atom displays a pyramidal geometry (Syed *et al.*, 2009), lying 0.27 (1) Å out of the plane of its substituents. The molecule adopts the general shape of a thick disc (as is reflected in the short a axis length), with the ester group folded under the aromatic ring (C1···C11 3.374 (2) Å, C11—S2···C2—C1 - 19.3 (1)°). The molecules are connected in chains parallel to the a axis by the classical hydrogen bond N—H01···O3 (H···O 2.30 (3) Å), supported by the "weak" hydrogen bonds H2···O1, H12···O1 (a bifurcated system) and H5C···S1 (H···*X* 2.66, 2.37, 2.88 Å respectively). Symmetry operators for all these H bonds involve a axis translation.

### Experimental

The title compound was synthesized by the reaction of methionine (0.02 mol) and 4-chlorobenzenesulfonyl chloride according to a reported procedure (Syed *et al.*, 2009). Recrystallization of the product from acetone/water afforded crystals suitable for X-ray analysis.

### Refinement

The NH hydrogen was refined freely. Methyl H atoms were located in difference syntheses, idealized to C—H 0.98 Å and H—C—H 109.5°, and refined as rigid groups allowed to rotate but not tip. Other H atoms were placed in calculated positions and refined using a riding model with C—H 0.95 Å for aromatic H and 1.00 Å for methine CH. Hydrogen *U* values were fixed at 1.5 × *U*(eq) of the parent atom for methyl H and 1.2 × *U*(eq) of the parent atom for other H. The compound is enantiomerically pure and its absolute configuration (*S* at C2) was confirmed by the Flack (1983) parameter. Data are 99.6% complete to 2θ 145°.

### Crystal data

**Table:**

C_12_H_16_ClNO_4_S_2_	*D*_x_ = 1.480 Mg m^−^^3^
*M**_r_* = 337.83	Melting point = 331–333 K
Orthorhombic, *P*2_1_2_1_2_1_	Cu *K*α radiation, λ = 1.54184 Å
*a* = 5.1814 (3) Å	Cell parameters from 10903 reflections
*b* = 12.6089 (8) Å	θ = 3.5–75.6°
*c* = 23.2137 (13) Å	µ = 4.92 mm^−^^1^
*V* = 1516.59 (16) Å^3^	*T* = 100 K
*Z* = 4	Tablet, colourless
*F*(000) = 704	0.20 × 0.12 × 0.06 mm

### Data collection

**Table:**

Oxford Diffraction Xcalibur Nova A diffractometer	3093 independent reflections
Radiation source: Nova (Cu) X-ray Source	3027 reflections with *I* > 2σ(*I*)
mirror	*R*_int_ = 0.033
Detector resolution: 10.3543 pixels mm^-1^	θ_max_ = 75.7°, θ_min_ = 3.8°
ω scans	*h* = −6→5
Absorption correction: multi-scan (*CrysAlis PRO*; Oxford Diffraction, 2008)	*k* = −15→15
*T*_min_ = 0.548, *T*_max_ = 1.000	*l* = −29→27
14469 measured reflections	

### Refinement

**Table:**

Refinement on *F*^2^	Secondary atom site location: difference Fourier map
Least-squares matrix: full	Hydrogen site location: inferred from neighbouring sites
*R*[*F*^2^ > 2σ(*F*^2^)] = 0.025	H atoms treated by a mixture of independent and constrained refinement
*wR*(*F*^2^) = 0.062	*w* = 1/[σ^2^(*F*_o_^2^) + (0.0339*P*)^2^ + 0.6444*P*] where *P* = (*F*_o_^2^ + 2*F*_c_^2^)/3
*S* = 1.04	(Δ/σ)_max_ = 0.001
3093 reflections	Δρ_max_ = 0.20 e Å^−^^3^
187 parameters	Δρ_min_ = −0.35 e Å^−^^3^
0 restraints	Absolute structure: Flack (1983), 1250 Friedel pairs
Primary atom site location: structure-invariant direct methods	Flack parameter: 0.005 (12)

### Special details

**Table:**

Geometry. All e.s.d.'s (except the e.s.d. in the dihedral angle between two l.s. planes) are estimated using the full covariance matrix. The cell e.s.d.'s are taken into account individually in the estimation of e.s.d.'s in distances, angles and torsion angles; correlations between e.s.d.'s in cell parameters are only used when they are defined by crystal symmetry. An approximate (isotropic) treatment of cell e.s.d.'s is used for estimating e.s.d.'s involving l.s. planes.
Refinement. Refinement of *F*^2^ against ALL reflections. The weighted *R*-factor *wR* and goodness of fit *S* are based on *F*^2^, conventional *R*-factors *R* are based on *F*, with *F* set to zero for negative *F*^2^. The threshold expression of *F*^2^ > σ(*F*^2^) is used only for calculating *R*-factors(gt) *etc*. and is not relevant to the choice of reflections for refinement. *R*-factors based on *F*^2^ are statistically about twice as large as those based on *F*, and *R*- factors based on ALL data will be even larger.

### Fractional atomic coordinates and isotropic or equivalent isotropic displacement parameters (Å^2^)

**Table:**

	*x*	*y*	*z*	*U*_iso_*/*U*_eq_	
C1	0.6060 (3)	0.70856 (13)	0.30125 (7)	0.0157 (3)	
C2	0.7658 (3)	0.64578 (13)	0.25767 (7)	0.0149 (3)	
H2	0.9529	0.6527	0.2676	0.018*	
C3	0.7198 (3)	0.69178 (13)	0.19720 (7)	0.0157 (3)	
H3A	0.5399	0.6770	0.1853	0.019*	
H3B	0.7427	0.7697	0.1984	0.019*	
C4	0.9040 (3)	0.64498 (13)	0.15292 (7)	0.0175 (3)	
H4A	0.8724	0.5677	0.1501	0.021*	
H4B	1.0834	0.6552	0.1665	0.021*	
C5	0.5616 (4)	0.65296 (18)	0.06022 (8)	0.0279 (4)	
H5A	0.5565	0.5761	0.0662	0.042*	
H5B	0.5334	0.6687	0.0194	0.042*	
H5C	0.4261	0.6868	0.0833	0.042*	
C6	0.5679 (4)	0.87023 (14)	0.34955 (8)	0.0267 (4)	
H6A	0.3949	0.8791	0.3332	0.040*	
H6B	0.6529	0.9395	0.3522	0.040*	
H6C	0.5541	0.8390	0.3881	0.040*	
C11	0.7949 (3)	0.48637 (13)	0.37042 (7)	0.0166 (3)	
C12	0.9359 (3)	0.56678 (13)	0.39648 (7)	0.0194 (3)	
H12	1.0801	0.5972	0.3774	0.023*	
C13	0.8635 (4)	0.60215 (14)	0.45083 (8)	0.0230 (3)	
H13	0.9576	0.6570	0.4694	0.028*	
C14	0.6511 (4)	0.55597 (14)	0.47751 (7)	0.0225 (3)	
C15	0.5128 (4)	0.47418 (15)	0.45226 (8)	0.0224 (4)	
H15	0.3716	0.4425	0.4719	0.027*	
C16	0.5837 (3)	0.43950 (13)	0.39798 (7)	0.0192 (3)	
H16	0.4898	0.3843	0.3796	0.023*	
O1	0.4034 (3)	0.67950 (10)	0.32058 (5)	0.0225 (3)	
O2	0.7177 (2)	0.80112 (10)	0.31298 (5)	0.0202 (3)	
O3	1.1274 (2)	0.47165 (9)	0.28709 (5)	0.0187 (2)	
O4	0.7538 (2)	0.34783 (10)	0.28792 (5)	0.0199 (3)	
S1	0.87182 (9)	0.70331 (3)	0.081959 (17)	0.02100 (10)	
S2	0.86006 (8)	0.45109 (3)	0.298219 (16)	0.01470 (9)	
Cl	0.55423 (10)	0.60320 (4)	0.54465 (2)	0.03195 (12)	
N	0.6935 (3)	0.53293 (11)	0.25852 (6)	0.0151 (3)	
H01	0.540 (6)	0.521 (2)	0.2616 (12)	0.041 (7)*	

### Atomic displacement parameters (Å^2^)

**Table:**

	*U*^11^	*U*^22^	*U*^33^	*U*^12^	*U*^13^	*U*^23^
C1	0.0189 (8)	0.0141 (7)	0.0140 (7)	0.0002 (7)	−0.0010 (7)	0.0023 (6)
C2	0.0149 (7)	0.0120 (7)	0.0176 (8)	−0.0009 (6)	−0.0006 (6)	−0.0011 (6)
C3	0.0160 (7)	0.0143 (7)	0.0168 (8)	0.0003 (6)	−0.0005 (6)	0.0005 (6)
C4	0.0157 (8)	0.0201 (8)	0.0168 (7)	0.0025 (6)	0.0004 (6)	0.0016 (6)
C5	0.0225 (9)	0.0396 (10)	0.0216 (9)	0.0020 (8)	−0.0013 (7)	0.0023 (8)
C6	0.0365 (11)	0.0214 (8)	0.0223 (8)	0.0063 (8)	0.0019 (8)	−0.0074 (7)
C11	0.0165 (8)	0.0140 (7)	0.0192 (8)	0.0022 (6)	−0.0011 (6)	0.0035 (6)
C12	0.0187 (8)	0.0184 (8)	0.0212 (8)	−0.0017 (6)	0.0012 (6)	0.0032 (6)
C13	0.0266 (9)	0.0195 (8)	0.0228 (8)	−0.0008 (8)	−0.0022 (8)	−0.0008 (7)
C14	0.0259 (9)	0.0222 (8)	0.0194 (8)	0.0043 (8)	0.0005 (7)	0.0026 (6)
C15	0.0211 (8)	0.0249 (8)	0.0211 (8)	−0.0006 (7)	0.0031 (7)	0.0068 (7)
C16	0.0189 (8)	0.0168 (8)	0.0219 (8)	−0.0030 (7)	−0.0019 (7)	0.0025 (6)
O1	0.0205 (7)	0.0197 (6)	0.0272 (6)	−0.0006 (5)	0.0076 (5)	−0.0008 (5)
O2	0.0254 (6)	0.0149 (6)	0.0204 (6)	−0.0018 (5)	0.0014 (5)	−0.0049 (5)
O3	0.0154 (5)	0.0161 (5)	0.0246 (6)	0.0007 (5)	0.0006 (5)	−0.0021 (4)
O4	0.0214 (6)	0.0126 (6)	0.0256 (6)	−0.0028 (5)	0.0000 (5)	−0.0004 (5)
S1	0.01868 (19)	0.0276 (2)	0.01674 (19)	0.00114 (18)	0.00273 (16)	0.00482 (15)
S2	0.01470 (18)	0.01144 (17)	0.01797 (18)	−0.00017 (15)	0.00043 (15)	0.00026 (13)
Cl	0.0416 (3)	0.0336 (2)	0.0206 (2)	0.0050 (2)	0.00561 (18)	−0.00285 (18)
N	0.0140 (6)	0.0128 (6)	0.0186 (7)	−0.0027 (5)	−0.0002 (5)	0.0000 (5)

### Geometric parameters (Å, °)

**Table:**

C1—O1	1.199 (2)	O4—S2	1.4337 (13)
C1—O2	1.331 (2)	S2—N	1.6306 (15)
C1—C2	1.528 (2)	C2—H2	1.0000
C2—N	1.472 (2)	C3—H3A	0.9900
C2—C3	1.538 (2)	C3—H3B	0.9900
C3—C4	1.522 (2)	C4—H4A	0.9900
C4—S1	1.8117 (17)	C4—H4B	0.9900
C5—S1	1.800 (2)	C5—H5A	0.9800
C6—O2	1.443 (2)	C5—H5B	0.9800
C11—C12	1.388 (2)	C5—H5C	0.9800
C11—C16	1.398 (2)	C6—H6A	0.9800
C11—S2	1.7667 (18)	C6—H6B	0.9800
C12—C13	1.390 (2)	C6—H6C	0.9800
C13—C14	1.391 (3)	C12—H12	0.9500
C14—C15	1.386 (3)	C13—H13	0.9500
C14—Cl	1.7422 (18)	C15—H15	0.9500
C15—C16	1.384 (2)	C16—H16	0.9500
O3—S2	1.4327 (13)	N—H01	0.81 (3)
			
O1—C1—O2	124.89 (16)	C2—C3—H3A	109.2
O1—C1—C2	124.31 (15)	C4—C3—H3B	109.2
O2—C1—C2	110.75 (14)	C2—C3—H3B	109.2
N—C2—C1	110.74 (13)	H3A—C3—H3B	107.9
N—C2—C3	109.73 (13)	C3—C4—H4A	108.9
C1—C2—C3	108.97 (13)	S1—C4—H4A	108.9
C4—C3—C2	111.92 (14)	C3—C4—H4B	108.9
C3—C4—S1	113.52 (11)	S1—C4—H4B	108.9
C12—C11—C16	121.41 (16)	H4A—C4—H4B	107.7
C12—C11—S2	119.77 (13)	S1—C5—H5A	109.5
C16—C11—S2	118.50 (13)	S1—C5—H5B	109.5
C11—C12—C13	119.20 (17)	H5A—C5—H5B	109.5
C12—C13—C14	118.92 (17)	S1—C5—H5C	109.5
C15—C14—C13	122.17 (17)	H5A—C5—H5C	109.5
C15—C14—Cl	118.93 (14)	H5B—C5—H5C	109.5
C13—C14—Cl	118.90 (14)	O2—C6—H6A	109.5
C16—C15—C14	118.88 (17)	O2—C6—H6B	109.5
C15—C16—C11	119.40 (16)	H6A—C6—H6B	109.5
C1—O2—C6	114.59 (14)	O2—C6—H6C	109.5
C5—S1—C4	101.18 (9)	H6A—C6—H6C	109.5
O3—S2—O4	120.36 (7)	H6B—C6—H6C	109.5
O3—S2—N	107.18 (7)	C11—C12—H12	120.4
O4—S2—N	106.09 (7)	C13—C12—H12	120.4
O3—S2—C11	108.08 (8)	C12—C13—H13	120.5
O4—S2—C11	108.27 (8)	C14—C13—H13	120.5
N—S2—C11	106.00 (7)	C16—C15—H15	120.6
C2—N—S2	118.99 (11)	C14—C15—H15	120.6
N—C2—H2	109.1	C15—C16—H16	120.3
C1—C2—H2	109.1	C11—C16—H16	120.3
C3—C2—H2	109.1	C2—N—H01	115.6 (19)
C4—C3—H3A	109.2	S2—N—H01	110.5 (19)
			
O1—C1—C2—N	−20.6 (2)	S2—C11—C16—C15	−173.17 (13)
O2—C1—C2—N	161.73 (13)	O1—C1—O2—C6	−3.3 (2)
O1—C1—C2—C3	100.19 (18)	C2—C1—O2—C6	174.33 (14)
O2—C1—C2—C3	−77.48 (16)	C3—C4—S1—C5	−69.77 (14)
N—C2—C3—C4	−67.63 (17)	C12—C11—S2—O3	29.75 (15)
C1—C2—C3—C4	170.97 (14)	C16—C11—S2—O3	−156.70 (13)
C2—C3—C4—S1	−176.06 (11)	C12—C11—S2—O4	161.63 (13)
C16—C11—C12—C13	−0.7 (3)	C16—C11—S2—O4	−24.82 (15)
S2—C11—C12—C13	172.62 (13)	C12—C11—S2—N	−84.90 (15)
C11—C12—C13—C14	−0.1 (3)	C16—C11—S2—N	88.66 (14)
C12—C13—C14—C15	1.5 (3)	C1—C2—N—S2	−94.83 (15)
C12—C13—C14—Cl	−177.99 (13)	C3—C2—N—S2	144.83 (12)
C13—C14—C15—C16	−2.0 (3)	O3—S2—N—C2	−47.30 (14)
Cl—C14—C15—C16	177.52 (14)	O4—S2—N—C2	−177.08 (12)
C14—C15—C16—C11	1.1 (3)	C11—S2—N—C2	67.96 (14)
C12—C11—C16—C15	0.3 (3)		

### Hydrogen-bond geometry (Å, °)

**Table:**

*D*—H···*A*	*D*—H	H···*A*	*D*···*A*	*D*—H···*A*
N—H01···O3^i^	0.81 (3)	2.30 (3)	3.1048 (18)	169 (3)
C2—H2···O1^ii^	1.00	2.66	3.637 (2)	166
C12—H12···O1^ii^	0.95	2.37	3.315 (2)	173
C3—H3B···O3^iii^	0.99	2.66	3.635 (2)	170
C5—H5B···S1^iv^	0.98	2.97	3.892 (2)	157
C5—H5C···S1^i^	0.98	2.88	3.665 (2)	138

Symmetry codes: (i) *x*−1, *y*, *z*; (ii) *x*+1, *y*, *z*; (iii) −*x*+2, *y*+1/2, −*z*+1/2; (iv) *x*−1/2, −*y*+3/2, −*z*.
